# BOR-Syndrome-Associated *Eya1* Mutations Lead to Enhanced Proteasomal Degradation of Eya1 Protein

**DOI:** 10.1371/journal.pone.0087407

**Published:** 2014-01-29

**Authors:** Amna Musharraf, Dagmar Kruspe, Jürgen Tomasch, Birgit Besenbeck, Christoph Englert, Kathrin Landgraf

**Affiliations:** Leibniz Institute for Age Research - Fritz Lipmann Institute e. V. (FLI), Jena, Germany; Montana State University, United States of America

## Abstract

Mutations in the human *EYA1* gene have been associated with several human diseases including branchio-oto (BO) and branchio-oto-renal (BOR) syndrome, as well as congenital cataracts and ocular anterior segment anomalies. BOR patients suffer from severe malformations of the ears, branchial arches and kidneys. The phenotype of *Eya1*-heterozygous mice resembles the symptoms of human patients suffering from BOR syndrome. The *Eya1* gene encodes a multifunctional protein that acts as a protein tyrosine phosphatase and a transcriptional coactivator. It has been shown that Eya1 interacts with Six transcription factors, which are also required for nuclear translocation of the Eya1 protein. We investigated the effects of seven disease-causing *Eya1* missense mutations on Eya1 protein function, in particular cellular localization, ability to interact with Six proteins, and protein stability. We show here that the BOR-associated *Eya1* missense mutations S454P, L472R, and L550P lead to enhanced proteasomal degradation of the Eya1 protein in mammalian cells. Moreover, Six proteins lead to a significant stabilization of Eya1, which is caused by Six-mediated protection from proteasomal degradation. In case of the mutant L550P, loss of interaction with Six proteins leads to rapid protein degradation. Our observations suggest that protein destabilization constitutes a novel disease causing mechanism for Eya1.

## Introduction

The human *EYA1* gene is a homolog of *Drosophila eya* (eyes absent), which is required for the development of the compound eye. In contrast to the single *eya* gene in *Drosophila*, mammals have four *Eya* paralogs (*Eya1-4*). The N-terminus of Eya proteins is required for function as co-activators of transcription [1], while the highly conserved C-terminal region called the Eya domain (ED) harbors an intrinsic phosphatase activity [2]–[4]. The Eya domain is also required for interaction with other proteins including Dach, Sipl1, the inhibitory G protein α subunit as well as Six proteins [5]–[8]. All Six family members are characterized by a homeobox DNA binding domain (HD) and a highly conserved Six domain (SD) that mediates protein-protein interaction with Eya [9]. Six proteins are bona fide transcription factors that induce nuclear translocation of Eya proteins [8].

Mutations in the human *EYA1* gene have been associated with several human diseases including branchio-oto (BO) and branchio-oto-renal (BOR) syndrome, as well as congenital cataracts and ocular anterior segment anomalies. BOR syndrome is an autosomal-dominant disorder characterized by branchial arch anomalies, hearing loss and kidney defects [10]–[12].

Knockout studies in mice revealed the physiological significance of *Eya1*. *Eya1* heterozygotes exhibit conductive hearing loss and renal abnormalities similar to those observed in BOR patients [13]. In contrast, *Eya1* homozygotes die at birth and show reduced head size and open eyelids, severe craniofacial and skeletal defects, absence of ears and kidney and other organs such as thymus and parathyroid [14]. Expression studies in mice have shown that *Eya1* is co-expressed with *Six1* and *Six2* in branchial arches, ear and kidney during development [15], [16]. Indeed, mutations in *Six* gene family members such as *SIX1* and *SIX5* also cause BOR syndrome while mutations in *SIX2* have been reported in patients with renal hypodysplasia, a phenotype observed in patients with BOR syndrome [17]–[19].

Analysis of the molecular mechanisms, by which mutations in *EYA1* lead to BOR syndrome, showed that several different aspects of Eya1 protein function can be affected, as for example the phosphatase activity, the interactions of Eya1 with Six, Dach, and Gα subunits, or both [20]–[25].

Here we have investigated the effects of seven disease causing *EYA1* mutations on various biological functions: cellular localization, ability to interact with Six proteins and protein stability. We show that Six proteins not only lead to the translocation of Eya1 to the nucleus but also to a significant stabilization of Eya1. Moreover, Eya1 is ubiquitinated and degraded by the proteasomal machinery, which is prevented by the interaction with Six proteins. Particularly one mutant, L550P did not interact with Six proteins and was highly unstable. Our observations suggest that protein destabilization constitutes a novel disease causing mechanism for Eya1.

## Experimental Procedures

### Expression constructs

Mammalian expression constructs for mouse *Eya1* were generated by cloning of the full-length cDNA from pHM6-Eya1 (kindly provided by Kyoshi Kawakami) into Rc/CMV (Invitrogen) including an N-terminal HA-tag for immunoblot analyses or pEGFP-C3 (BD Biosciences) for fluorescence microscopy. *Eya1* missense mutations were introduced as reported previously [21]. *Eya1* deletion constructs for *in vivo* ubiquitination assays were generated by site-directed mutagenesis (QuikChange, Stratagene) using specific primers (Table S1). Expression vector containing human *SIX1* coding sequence was a kind gift from E.A. Otto. FLAG tagged mouse *Six2* was constructed as described [26]. Plasmids pHis-Ubiquitin and pH-Ras were generous gifts from M. Treier and I. Rubio, respectively.

### Cell culture and transfections

COS-7, U2OS and MK4 cells [27] were maintained in Dulbecco Modified Eagle's Medium containing 10% fetal bovine serum. Cells were transfected using Superfect (Qiagen) according to the manufacturer. For MK4 stable cells pBabe Puro plasmid [28] was transfected with a *Six* expression construct in the ratio of 1:10. Cells were selected with 1 µg/ml puromycin for 4 days. MK4 cells were transfected with *Eya1*-specific siRNA (5′-CCGAGGCAGAAGAAACAATAA-3′, Qiagen) using siLentFect (Bio-Rad).

### Generation of Eya1 antibodies

An N-terminal fragment of Eya1 (aa236-305) was cloned into pGEX-KG and transformed into *E. coli* BL21. Recombinant GST fusion protein was isolated from bacteria as described [21], and used for rabbit immunization (ImmunoGlobe). Antisera were affinity-purified on columns containing immobilized Eya1-N-terminus.

### Immunocytochemistry

COS-7 cells were grown on cover slips and transfected with plasmids of interest. 36 h later, cells were fixed in 4% PFA. FLAG-Six2 was detected using anti-FLAG antibody (rabbit polyclonal, Sigma) and rhodamine-conjugated goat anti-rabbit antibody (Jackson ImmunoResearch). The cell nucleus was stained with Hoechst dye (Sigma). After mounting with glycergel (DAKO), cells were visualized using a fluorescence microscope (Zeiss).

### Protein extraction and immunoblot analyses

Preparation of nuclear and cytoplasmic extracts was performed according to Weih *et al*. [29]. For whole cell extracts, cells were lysed in lysis buffer (25 mM Hepes pH 7.9, 1 mM EDTA, 150 mM NaCl, 1% NP-40 and cØmplete (Roche) proteinase inhibitor). Equivalent amount of protein was resolved by 10% SDS-PAGE and immunoblotting using anti-HA 6E2 antibody (Cell Signaling) for detection of Eya1 and anti-FLAG M2 antibody (Sigma) for detection of Six proteins. To determine the purity of the nuclear and cytoplasmic fractions, the membranes were probed with anti- c-jun or anti- Bcl-2 antibodies (Santa Cruz Biotechnology). Equal loading was confirmed by detection of β-actin (A5316, Sigma).

### Co-immunoprecipitation

Whole cell lysate was preincubated with Protein G Sepharose (Calbiochem) for 1 h at 4°C. After centrifugation at 2,000 rpm for 2 min, the lysate was mixed with anti-FLAG antibody overnight at 4°C. Afterwards, the complex was allowed to attach to the Protein G sepharose for 2–3 h. Immunocomplexes were washed with lysis buffer, and analysed via SDS-PAGE and immunoblotting

### In Vivo Ubiquitination Assay

COS-7 cells were transfected with 5 µg of His-Ubiquitin and other expression plasmids as indicated. 24 h post-transfection cells were treated with proteasomal inhibitor MG132 (10 µM) for another 24 h. Cells were harvested by centrifugation, and His-ubiquitin-labelelled proteins were purified according to Pan *et al*. [30]. Ubiquitinated Eya1 was detected by immunoblotting using anti-Eya1 or anti-HA antibody.

## Results

### Eya1 mutant L550P fails to translocate into the nucleus in presence of Six proteins

**Figure 1:**
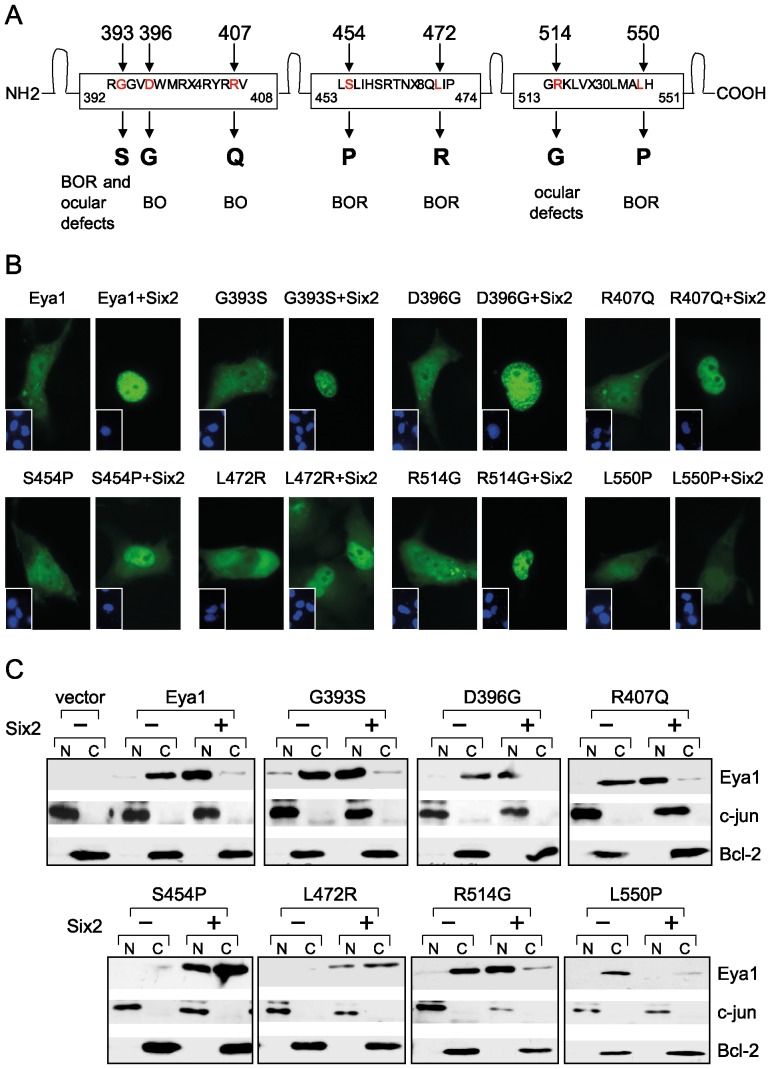
The BOR mutant L550P fails to translocate into the nucleus in presence of Six. (A) Eya domain of human Eya1 depicting the disease associated missense mutations included in this study. BOR branchio-oto-renal; BO branchio-otic; OD ocular defects. (B) Cellular localization of Eya1 and disease-associated Eya1 mutants. COS-7 cells were transfected with expression plasmids encoding EGFP-Eya1 or mutants either alone or in combination with Six2. Insets show counterstaining with Hoechst dye. (C) COS-7 cells were transfected with HA-tagged *Eya1* or mutants together with FLAG-Six2 or empty vector. Nuclear (N) and cytoplasmic (C) extracts were analyzed by immunoblotting.

We addressed a potential impact of selected BO/BOR associated missense mutations on the subcellular localization of the Eya1 protein. All these mutations reside in the conserved Eya domain (ED) and lead to substitutions of amino acids that are conserved in human and mouse Eya1 (Fig. 1A). We introduced the disease causing *Eya1* mutations in an EGFP-*Eya1* encoding construct and analyzed the localization of the fusion proteins in COS-7 cells. All mutant proteins were present in the cytoplasm similar to wild type Eya1 suggesting that these mutations do not alter the subcellular localization of Eya1 protein *per se* (Fig. 1B).

As described previously [8], the wild type Eya1 fusion protein was quantitatively translocated into the nucleus upon coexpression with Six2 (Fig. 1B). Mutant Eya1 proteins G393S, D396G, R407Q and R514G translocated into the nucleus with efficiency similar to that of wild type Eya1. In contrast, S454P and L472R showed intense staining in the nucleus and weaker staining in the cytoplasm thus exhibiting less ability for efficient translocation. The mutant L550P showed no staining in the nucleus in presence of Six2 protein suggesting that this mutation in Eya1 has rendered the protein incapable of translocation.

We further analyzed the intracellular distribution of Eya1 and mutants in absence and presence of Six2 by cell fractionation and immunoblot analyses. Eya1 and mutants alone were present in the cytoplasm while in presence of Six2 a major fraction of wild type and mutant proteins were detected in the nucleus (Fig. 1C). However, mutants S454P and L472R were not as efficiently translocated from cytoplasm into the nucleus as the wild type Eya1 protein while localization of Six2 was not altered in presence of wild type or mutant Eya1 (Fig. S1). The mutant L550P did not show any translocation in presence of Six2.

### Six proteins stabilize Eya1 except for the L550P mutant

**Figure 2:**
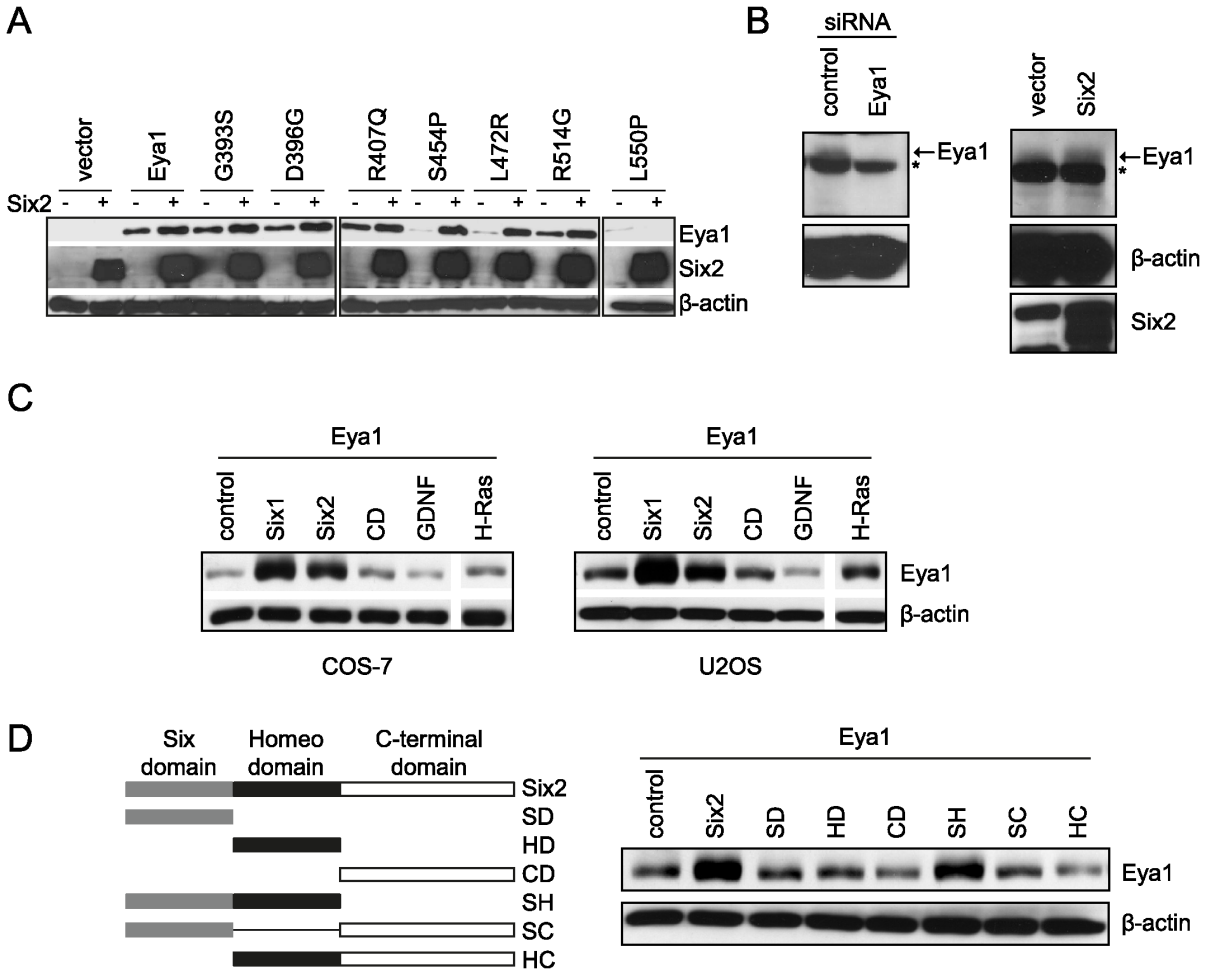
Six proteins stabilize Eya1 and Eya1 mutants with the exception of L550P. (A) COS-7 cells were transfected with empty vector (control), HA-Eya1 or mutants, and FLAG-Six2. Equal amounts of protein were analyzed by SDS-PAGE and immunoblotting using anti-HA and anti-FLAG antibody. (B) Endogenous Eya1 is stabilized in presence of Six2. MK4 cells were transfected with *Eya1*-specific or non-target control siRNA and Eya1 protein levels were detected by immunoblotting using anti-Eya1 antibody (left). MK4 cells stably expressing FLAG-Six2 were subject to immunoblot analyses using Eya1-specific antibody (right). Asterisk indicates unspecific signal. (C) Six induces accumulation of Eya1 protein in different cell lines via the conserved Six and homeo domain. COS-7 and U2OS cells were transfected with HA-*Eya1* and expression vectors for *Six1*, *Six2*, C-terminal domain of Six2 (CD), *GDNF* and *H-Ras*. Eya1 protein levels were detected by immunoblotting using anti-HA antibody. (D) Schematic representation of the Six2 deletion constructs used for characterization of Eya1 stabilization (left). COS-7 cells were co-transfected with HA-Eya1, Six2 or deletion constructs as indicated. Eya1 protein levels were analyzed by immunoblotting.

The comparison of Eya1 protein levels in the absence and presence of Six2 suggested that Eya1 might be stabilized by Six protein. To assess this possibility, COS-7 cells were co-transfected with constructs encoding HA-Eya1 or the respective mutant proteins as well as FLAG-Six2. In presence of Six2, we observed significantly increased Eya1 protein levels (Fig. 2A). Only Eya1 mutant L550P did not show enhanced protein levels in presence of Six2. Furthermore, we examined the effect of Six2 on endogenous Eya1. For this we generated an Eya1-specific antibody and confirmed its specificity in MK4 cells transfected with an Eya1-specific siRNA (Fig. 2B *left*). After transfection of MK4 cells with FLAG-Six2 there was an increase in endogenous Eya1 protein levels suggesting that endogenous Eya1 protein was also stabilized by Six protein (Fig. 2B *right*).

### Six stabilizes the Eya1 protein via the conserved Six and homeo domain

To address the specificity of the observed Eya1 stabilization we performed co-expression studies in COS-7 and U2OS cells. For this, we co-transfected the cells with expression constructs for *Eya1* and *Six1*, *Six2*, the C-terminal domain of Six2 (CD), *GDNF* or *H-Ras*, and analysed Eya1 protein levels. There was a robust increase in Eya1 protein levels only in presence of Six1 or Six2 in both cell lines (Fig. 2C). To determine which region of Six protein mediates stabilization of Eya1, several Six2 deletion fragments (Fig. 2D) were co-expressed with *Eya1*. Eya1 protein levels were enhanced in presence of Six2 full-length as well as a deletion fragment including both Six domain and homeo domain (SH). In contrast, all other expression plasmid failed to increase Eya1 levels. Thus, the Six and homeo domains of Six2 are required for Eya1 stabilization.

### Six protects Eya1 from degradation via the ubiquitin-proteasomal pathway

**Figure 3:**
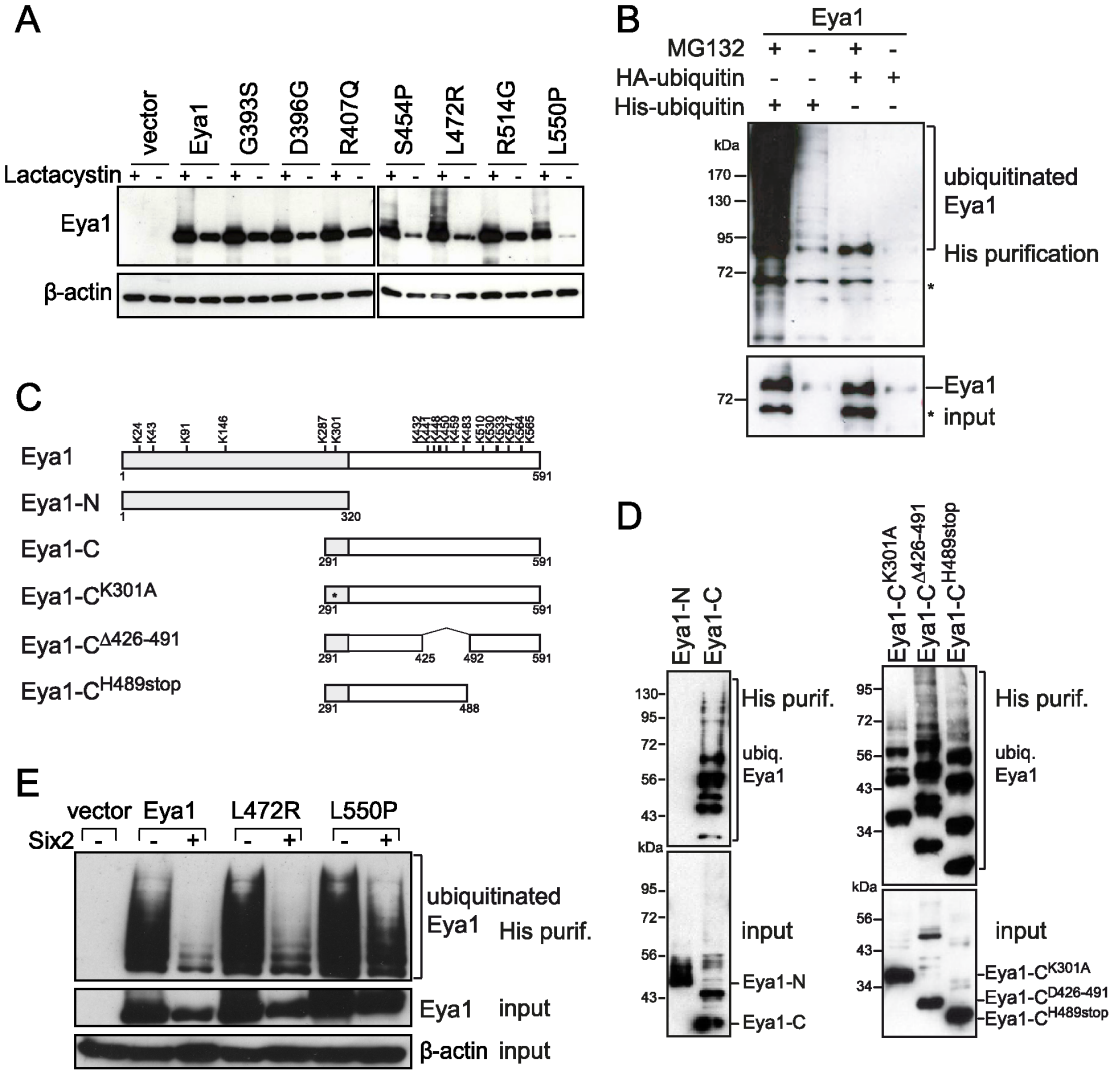
Eya1 degradation occurs via the proteasome and is inhibited by Six proteins. (A) Eya1 and its mutants accumulate in the presence of the proteasome inhibitor lactacystin. COS-7 cells were transfected with wild type or mutant HA-Eya1 or empty vector. 24 h post-transfection cells were treated with lactacystin (1 µM). Cells were lysed and Eya1 was detected by immunoblotting. (B) Eya1 is ubiquitinated. COS-7 cells were co-transfected with HA-Eya1 and HA-ubiquitin or His-ubiquitin. 24 h later, cells were treated with MG132 (10 µM) or DMSO for 24 h. For *in vivo* ubiquitination assay, His-ubiquitin-labelled proteins were purified from cell lysates followed by immunoblotting and detection of Eya1 using anti-Eya1 antibody. Asterisks indicate unspecific signals. (C) Schematic representation of the Eya1 mutants used for *in vivo* ubiquitination assay. Position of lysine residues is indicated in the scheme of full-length Eya1. (D) Eya1 ubiquitination occurs in two distinct regions of the Eya domain. COS-7 cells were co-transfected with His-ubiquitin and the indicated *Eya1* expression constructs, treated with MG132 (10 µM), and subjected to *in vivo* ubiquitination assay followed by immunoblotting and detection of Eya1 using anti-HA antibody. (E) Six proteins inhibit ubiquitination of Eya1. COS-7 cells overexpressing wild type or mutant HA-Eya1, His-ubiquitin and Six2 or empty vector were subjected to *in vivo* ubiquitination assay. Ubiquitinated Eya1 protein was detected by immunoblotting using anti-HA antibody. β-actin was used as a loading control for total cell lysate.

We next addressed whether the instability of the disease-associated Eya1 mutants S454P, L472R, and L550P is caused by an enhanced degradation via the proteasomal pathway. Treatment of COS-7 cells overexpressing *Eya1* or the BOR-associated mutant proteins with the proteasome inhibitor lactacystin led to the accumulation of the Eya1 protein indicating that Eya1 is degraded via the proteasome (Fig. 3A). Moreover, the three BOR-associated Eya1 mutants S454P, L472R and L550P accumulated to a similar amount as the wild type protein in lactacystin-treated cells. This indicated that instability of the mutants S454P, L472R and L550P was mediated by enhanced degradation via the proteasome. To show that Eya1 indeed is ubiquitinated, an *in vivo* ubiquitination assay was performed. The level of His-ubiquitin-labelled Eya1 was drastically enhanced in presence of MG132 confirming that Eya1 is a target of the proteasomal pathway (Fig. 3B). To determine which part of Eya1 is ubiquitinated, an ubiquitination assay was performed using N- or C-terminal fragments of Eya1 (Fig. 3C,D). His-ubiquitin-labeled proteins could only be purified from cells overexpressing the C-terminal domain of Eya1, but not from cells overexpressing the N-terminal part of Eya1, indicating that ubiquitination of Eya1 occurs in the conserved Eya domain (Fig. 3D left). The Eya domain contains 12 lysine residues, which can be targets for ubiquitination. To further narrow down the ubiquitination site, different chimeric proteins were generated: a deletion of overall 66 amino acids including a cluster of 6 lysine residues in the middle part of the Eya1 C-terminus (Δ426–491), and a deletion of the C-terminally located 103 amino acids including 6 lysine residues (H489stop; Fig. 3C). Both deletions did not affect ubiquitination of Eya1 indicating that ubiquitination occurs in two distinct regions of the Eya domain (Fig. 3D right).

We next asked, whether Six proteins were enhancing the Eya1 or the Eya1 mutant protein levels by preventing them from proteasomal degradation. Indeed, there was a dramatic reduction in ubiquitination of Eya1 and the mutant L472R in presence of Six2 (Fig. 3E). In the case of L550P ubiquitination levels were still comparable to those of Eya1 alone even in presence of Six2 suggesting that Six2 could not prevent degradation of L550P via the proteasomal machinery. This suggests that Six regulates Eya1 protein stability by suppressing its ubiquitination and degradation.

### Stabilization of Eya1 by Six is dependent on protein-protein interaction

**Figure 4:**
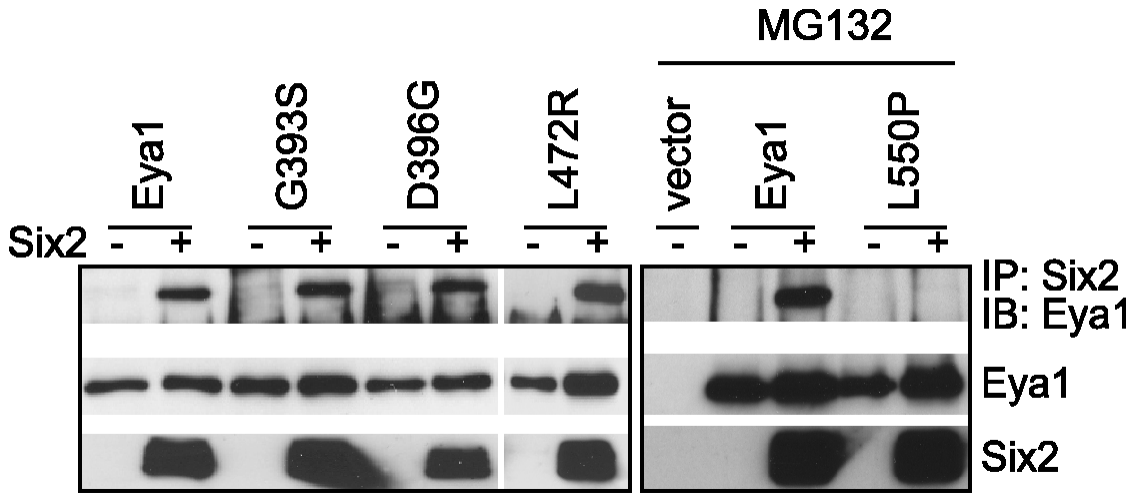
Eya1 stabilization requires interaction with Six proteins. COS-7 cells were transfected with HA-Eya1 or mutants and FLAG-Six2 as indicated (left). In case of the mutant L550P cells were treated with MG132 (10 µM) to enhance protein levels (right). Six2 was immunoprecipitated using anti-FLAG antibody, and interacting Eya1 was detected by immunoblotting.

Since Eya1 was shown to interact directly with Six transcription factors via its conserved Eya domain, we examined whether the stabilization of Eya1 by Six proteins required an interaction between these proteins. We also included the Eya1 mutants G393S, D396G, L472R and L550P in this experiment. COS-7 cells were co-transfected with expression plasmid encoding either full length Eya1 or the mutant proteins and Six2 expression plasmid or empty vector and examined the interaction by co-immunoprecipitation and subsequent immunoblotting. Since the L550P mutant protein is highly unstable and diminishes rather quickly, we treated the cells 24 h after transfection with the proteasomal inhibitor MG132 for additional 24 h. Wild type Eya1 and mutant proteins were efficiently co-immunoprecipitated by Six2. In spite of the enhanced protein levels after treatment with the proteasomal inhibitor, the mutant L550P failed to bind Six2 (Fig. 4). In summary, the Eya1 mutant L550P which is not stabilized by Six2 also fails to interact with Six2 indicating that stabilization of Eya1 requires an interaction between these proteins. The observations that we report here suggest that failure of Eya1 to interact with Six proteins renders the former susceptible to proteasome-mediated degradation.

## Discussion

In order to understand the molecular mechanisms by which mutations in the human *EYA1* gene lead to BOR/BO syndrome, we have analyzed the functional importance of Eya1 domain specific missense mutations. We have, therefore, included a variety of missense mutations in our study that result in BOR syndrome, BO syndrome or ocular defects.

As a first step, we investigated whether the *Eya1* missense mutations alter the subcellular localization of the protein and if they affect the nuclear translocation of Eya1 by Six. The data presented here show that all mutant proteins were present in the cytoplasm similar to wild type Eya1. Interestingly, in the presence of Six2 all mutants except of L550P were able to translocate into nucleus. L550P did not translocate from the cytoplasm to the nucleus in the presence of Six2. However, in the presence of Six2 there were mutants (G393S, D396G, R407Q and R514G) that displayed efficient nuclear translocation similar to the wild type while other mutants (S454P, L472R) demonstrated only weak ability to translocate into the nucleus. These results are in line with a previous study by Buller *et al*. who showed that the Eya1 mutants G393S, S454P, L472R and R514G do not prevent nuclear translocation when each construct was transfected with Six1 or Six2 [20]. Interestingly, localization of Six2 protein was not affected by the mutants S454P or L472R, which are sequestered in the cytoplasm, indicating that Six2 does not interact with mutant Eya1 proteins in the cytoplasm.

Several observations in this study provide evidence that Eya1 protein levels are stabilized by Six protein and this stabilization requires interaction between Eya1 and Six proteins. We have demonstrated that Eya1 is ubiquitinated and degraded via the proteasomal machinery. This is in line with a recent report that demonstrated regulation of Eya1 levels by ubiquitin-mediated proteolysis [31]. Furthermore, Eya1 ubiquitination can be suppressed by Six2. Interestingly, Six proteins bind to the conserved Eya domain of Eya1 where also ubiquitination of the protein has been demonstrated to occur. Thus, it is possible that interaction with Six prevents Eya1 ubiquitination because an important ubiquitination site is not accessible. Another explanation could be that ubiquitination and subsequent degradation of Eya1 exclusively occur in the cytoplasm of the cell and nuclear translocation of Eya1 by Six proteins sequesters the protein away from the place of ubiquitination. Evidence for the latter hypothesis is provided by the observation that both Six- and homeo-domain of Six2 are necessary to stabilize Eya1. Both six- and homeo-domain have also been previously described to be essential for nuclear translocation of Eya, whereas for interaction with Eya the Six domain alone is sufficient [8], [32].

Interestingly, the Eya1 mutant L550P analyzed in this work is rapidly degraded upon synthesis and not prevented from ubiquitination and thus degradation in presence of Six2. L550P is a BOR associated mutant and impaired translocation, stabilization or interaction could be the reason for the disease in this case. The two BOR-type Eya1 mutants, S454P and L472R, also lead to instability of Eya1 protein in absence of Six2, while interaction with Six2 and Six2-induced nuclear translocation was still detectable [20]. It was proposed recently that both these mutations lead to conformational changes in Eya1 protein structure and loss of transactivation activity of the Eya-Six complex [23]. Interestingly, BOR patients harboring the different mutations, S454P, L472R, or L550P, do not show obvious differences in the BOR-syndrome related phenotype [33], [34]. Hence, S454P and L472R might behave like dominant-negative mutants and compete with wild type Eya1 for interaction with Six2, thereby perturbing transcription of target genes. In contrast, the mutant G393S, which is derived from a patient with combined BOR syndrome and ocular defects did not affect any of the Eya1 protein characteristics addressed in this work and in previous studies [23], [25]. Similar results were obtained for the mutants D396G, R407Q and R514G, which are associated with BO syndrome and ocular defects, respectively. Eya1 is a multifunctional protein and a possible target of RTK signaling [35]. Furthermore, Eya1 has recently been shown to be involved in controlling cell polarity, cell fate, orientation of the mitotic spindle and notch signaling [36]. Hence, these mutations may compromise some other unknown or some or all of these recently identified functions of Eya1.

In line with our data, Patrick *et al*. recently reported that both Six1 and Six2 are also stabilized in presence of Eya2 and Eya1, respectively [24]. The fact that Eya1 and Six1 or Six2 are co-expressed in several organs suggests that these proteins might regulate the stability of each other *in vivo*. The consequence of stabilization of Eya1 and/or Six proteins may provide a platform for recruiting tissue specific factors to control differentiation of those tissues. Recently, it has been reported that Pax6 [37] and Six1 [38] are degraded by the proteasomal machinery. Together with the observation that we report here it seems that ubiquitination and degradation is a common mechanism that regulates activity of all members of the Pax/Eya/Six organ specification network [26] to control target gene expression. Finally, our data suggest that impaired Eya1 stabilization is a novel disease causing mechanism in BOR- and related syndromes.

## Supporting Information

Figure S1 **Localization of Six2 protein is not altered in presence of mutants S454P and L472R compared to wild type Eya1.** (A) Cellular localization of Eya1 or disease-associated Eya1 mutants and Six2. COS-7 cells were transfected with expression plasmids encoding EGFP fusion proteins of wild type Eya1 or Eya1 mutants S454P and L472R, in combination with a FLAG-Six2 encoding plasmid. FLAG-Six2 was detected by immunofluorescence using anti-FLAG antibody. (B) COS-7 cells were transfected with wild type Eya1 or Eya1 mutants as indicated together with FLAG-Six2. Nuclear (N) and cytoplasmic (C) extracts were analyzed by immunoblotting using anti-FLAG antibody for detection of FLAG-Six2.
(TIF)

Table S1 Primer sequences for generation of *Eya1* mutations for *in vivo* ubiquitination assays.
(DOC)
