# Supplementary figures and images for: BOR-Syndrome-Associated Eya1 Mutations Lead to Enhanced Proteasomal Degradation of Eya1 Protein
Source: PLoS One. 2014 Jan 29;9(1):e87407. doi: 10.1371/journal.pone.0087407 (PMC3906160; doi:10.1371/journal.pone.0087407)

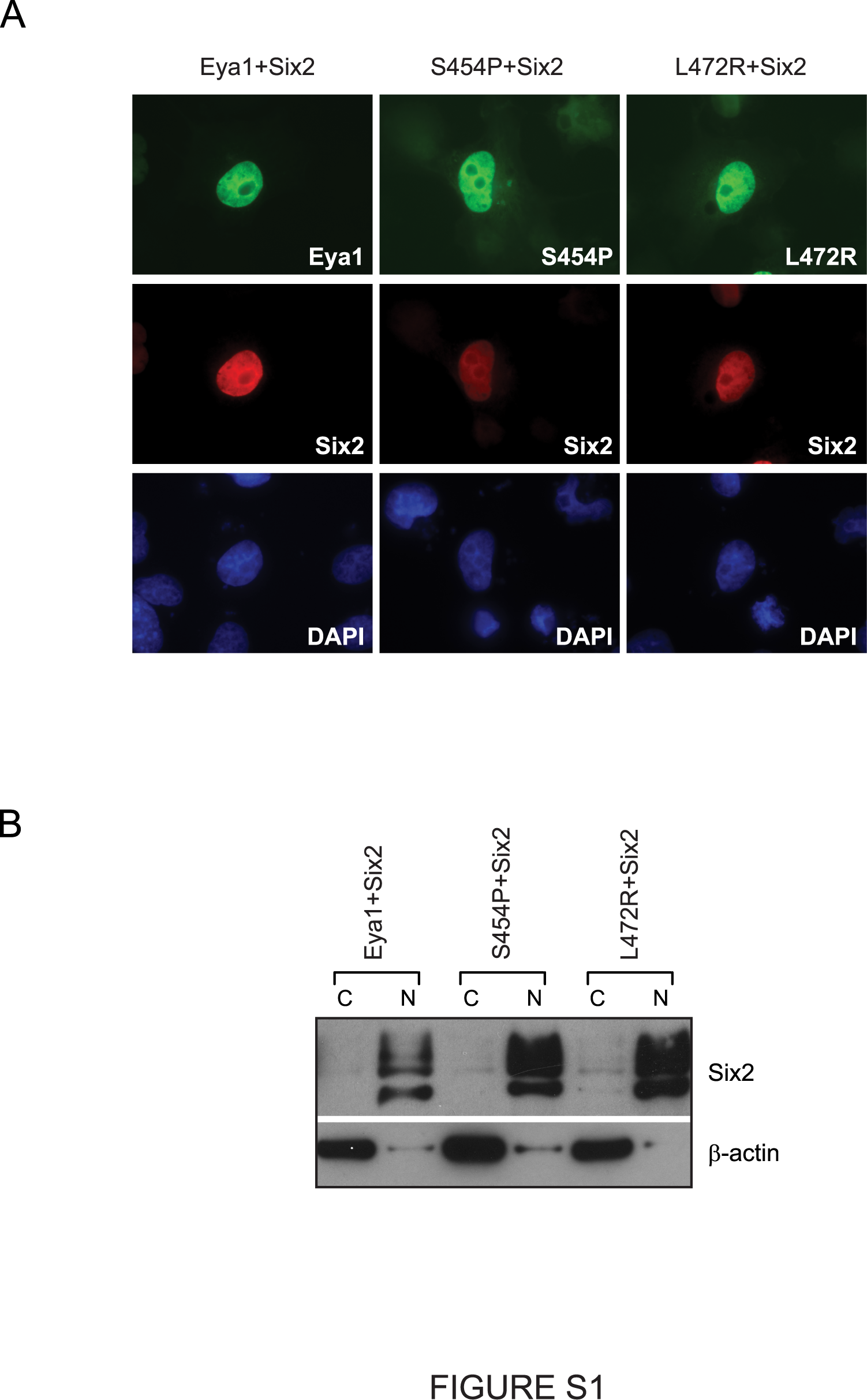

Supplement: Figure S1 — Localization of Six2 protein is not altered in presence of mutants S454P and L472R compared to wild type Eya1. (A) Cellular localization of Eya1 or disease-associated Eya1 mutants and Six2. COS-7 cells were transfected with expression plasmids encoding EGFP fusion proteins of wild type Eya1 or Eya1 mutants S454P and L472R, in combination with a FLAG-Six2 encoding plasmid. FLAG-Six2 was detected by immunofluorescence using anti-FLAG antibody. (B) COS-7 cells were transfected with wild type Eya1 or Eya1 mutants as indicated together with FLAG-Six2. Nuclear (N) and cytoplasmic (C) extracts were analyzed by immunoblotting using anti-FLAG antibody for detection of FLAG-Six2. (TIF) [file pone.0087407.s001.tif]
