# Supplementary material for: BOR-Syndrome-Associated Eya1 Mutations Lead to Enhanced Proteasomal Degradation of Eya1 Protein
Source: PLoS One. 2014 Jan 29;9(1):e87407. doi: 10.1371/journal.pone.0087407 (PMC3906160; doi:10.1371/journal.pone.0087407)
Supplement: Table S1 — Primer sequences for generation of Eya1 mutations for in vivo ubiquitination assays. (DOC) [file pone.0087407.s002.doc]

**Table S1. Primer sequences for generation of *Eya1* mutations for *in vivo* ubiquitination assays**

| **Mutation** | **Forward Primer** | **Reverse Primer** |
| --- | --- | --- |
| K301A | CGAGGTTCAGATGGGGCGTCACGTGGCCGA | TCGGCCACGTGACGCCCCATCTGAACCTCG |
| Δ426-491f | ACTGGTGTCCGAGGTACGAACTGTGTGAAT | ATTCACACAGTTCGTACCTCGGACACCAGT |
| H489stop | GCCCTCTCCCTCATCTAGTCCCGGACGAAC | GTTCGTCCGGGACTAGATGAGGGAGAGGGC |

Forward and reverse primers are given in 5´-3´direction.
